# The effects of different fractions of *Coriandrum sativum* on pentylenetetrazole-induced seizures and brain tissues oxidative damage in rats

**Published:** 2016

**Authors:** Akbar Anaeigoudari, Mahmoud Hosseini, Reza Karami, Farzaneh Vafaee, Toktam Mohammadpour, Ahmad Ghorbani, Hamid Reza Sadeghnia

**Affiliations:** 1 *Department of Physiology, School of Medicine, Jiroft University of Medical Sciences, Jiroft, Iran*; 2 *Neurogenic Inflammation Research Center & Department of Physiology, School of Medicine, Mashhad University of Medical Sciences, Mashhad, Iran*; 3 *Student Research Committee, School of Medicine, Mashhad University of Medical Sciences, Mashhad, Iran *; 4 *Neurocognitive Research Center & Department of Pharmacology, School of Medicine, Mashhad University of Medical Sciences, Mashhad, Iran*; 5 *Pharmacological Research Center of Medicinal Plants, School of Medicine, Mashhad University of Medical Sciences, Mashhad, Iran*

**Keywords:** *Coriandrum sativum*, *Fractions*, *Seizures*, *Pentylenetetrazole*, *Oxidative stress*

## Abstract

**Objective:**

In the present work, the effects of different fractions of *Coriandrum sativum (C. sativum)*, on pentylenetetrazole (PTZ)-induced seizures and brain tissues oxidative damage were investigated in rats.

**Materials and Methods:**

The rats were divided into the following groups: (1) vehicle, (2) PTZ (90 mg/kg), (3) water fraction (WF) of* C. sativum* (25 and 100 mg/kg), (4) *n*-butanol fraction (NBF) of* C. sativum* (25 and 100 mg/kg), and (5) ethyl acetate fraction (EAF) of* C. sativum* (25 and 100 mg/kg).

**Results:**

The first generalized tonic-clonic seizures (GTCS) latency in groups treated with 100 mg /kg of WF or EAF was significantly higher than that of PTZ group (p<0.01). In contrast to WF, the EAF and NBF were not effective in increasing the first minimal clonic seizure (MCS) latency. Malondialdehyde (MDA) levels in both cortical and hippocampal tissues of PTZ group were significantly higher than those of control animals (p<0.001). Pretreatment with WF, NBF, or EAF resulted in a significant reduction in the MDA levels of hippocampi (p<0.01 - p<0.001). Following PTZ administration, a significant reduction in total thiol groups was observed in the brain tissues (p<0.05). Pretreatment with WF and NBF significantly elevated thiol concentrations in cortical and hippocampal tissues, respectively (p<0.05).

**Conclusion:**

The present study showed that different fractions of *C. sativum* possess antioxidant activity in the brain and WF and EAF of this plant have anticonvulsant effects.

## Introduction

Epilepsy is a common neurological disease which affects approximately 1% of the population (Sander, 2003 ▶). It is characterized by abnormal episodic bursts of electrical activity in neurons which has significant impact on the cognitive processes and behavior of the affected patients (Meador, 2002 ▶). Accumulating evidence revealed an important role for oxidative stress both as a consequence and a cause of epileptic seizures (Golechha et al., 2010 ▶; Patel, 2004 ▶). It has been found that prolonged seizures resulted in oxidative damage to lipids, DNA, and susceptible proteins (Kudin et al., 2002 ▶). Moreover, increased amounts of free radicals have been obsereved during seizures (Gupta and Briyal, 2006 ▶).

 The central nervous system (CNS) tissues contain high levels of fatty acids (Halliwell, 1992 ▶). It has been shown that membrane lipid constituents in the CNS are highly susceptible to oxidative injury (Coyle and Puttfarcken, 1993 ▶; Frolich and Riederer, 1995 ▶). 

Oxidative damage plays an important role in the pathogenesis of various CNS disorders and neurobehavioral impairments (Qureshi et al., 2004 ▶; Sharma et al., 2009 ▶). Also, it has been well documented that functional impairments which occur in central nervous system during epilepsy and seizure may be at least in part due to brain tissues oxidative damages (Mehla et al., 2010 ▶; Parfenova et al., 2012 ▶). Furthermore, the anti-convulsant activity of several agents with antioxidant properties such as melatonin, vineatrol, trans-resveratrol and alpha lipoic acid has been documented in studies (Gupta and Briyal, 2006 ▶; Sharma et al., 2005 ▶). Consistent with these observations, there are some reports showing that reactive oxygen species (ROS) may underlie the convulsant and neurotoxic effects of pentylenetetrazol (PTZ) (Aldarmaa et al., 2010 ▶). Collectively, it appears that a promising approach to provide neuroprotection against epilepsy or to find new therapeutics for this disorder is the use of antioxidants. 

Medicinal plants are good sources of new therapeutics for human diseases. *Coriandrum sativum *(*C. sativum*), an annual herb, belonging to the Apiaceae family, has been reported to have a wide range of biological activities including sedative-hypnotic, anti-diabetic, hypolipidemic, and hepatoprotective effects (Rakhshandeh et al., 2012 ▶; Eidi et al., 2009 ▶; Dhanapakiam et al., 2008 ▶; Samojlik et al., 2010 ▶). Experimental studies have also revealed strong antioxidant activity of *C. sativum* that is superior to known antioxidants like ascorbic acid (Samojlik et al., 2010 ▶; Hashim et al., 2005 ▶; Misharina and Samusenko, 2008 ▶; Sultana et al., 2010 ▶). In our previous works, we found that aerial parts of *C. sativum* has sedative effect and also their water-soluble compound(s) induces neuroprotective activity under stressful conditions like ischemia (Rakhshandeh et al., 2012 ▶, Ghorbani et al., 2011 ▶). Emam Ghoreyshi et al. reported that the seed of* C. sativum* possess anti-convulsant activity (Emam Ghoreyshi et al., 2008 ▶). However, so far, no study has been done to evaluate anti-convulsant activity of aerial parts of *C. sativum*. Since the aerial parts, but not the seeds, of this plant are widely used as vegetable all over the world, therefore it is reasonable to investigate anti-convulsant activity of these parts of *C. sativum*. 

PTZ is a selective inhibitor of the chloride channel which is coupled to the GABAA receptor (Sejima et al., 1997 ▶). It is a well-known chemo-convulsant which is frequently used for evaluation of anti-epileptic properties (Porter et al., 1984 ▶; Hosseinzadeh and Sadeghnia, 2007 ▶). A high dose of PTZ induces a continued seizure activity which progresses from mild myoclonic jerks to face and forelimbs clonus without loss of righting reflex (which is known as minimal clonic seizure, MCS), to clonic seizures of limbs with loss of righting reflex, to full tonic extension of both forelimbs and hindlimbs (generalized tonic-clonic seizures, GTCS) (Loscher et al., 1991 ▶). PTZ has been repeatedly used at the doses of 90-100 mg/ kg to induce MCS and GTCS seizures (Hosseini et al., 2011 ▶; Hosseini et al., 2009 ▶; Ebrahimzadeh Bideskan et al., 2011 ▶; Hosseini et al., 2012 ▶; Hosseini et al., 2013 ▶). 

In our previous work, we found that the hydro-alcoholic extract and fractions of *C. sativum *induced sedative/hypnotic effects at the doses of 50, 100, 200 and 400 mg/kg (Rakhshandeh et al., 2012 ▶). In the present study, we aimed to evaluate the possible protective effects of water fraction (WF), *n*-butanol fraction (NBF) and ethyl acetate fraction (EAF) of* C. sativum* on brain tissues oxidative damage in PTZ-induced seizures model in rat. Behavioral responses to PTZ administration were also evaluated in animals treated with these fractions.

## Materials and Methods

**Drugs and chemicals**

PTZ was purchased from Sigma Aldrich Company (St. Louis, USA). Other chemical compounds such as thiobarbituric acid (TBA), trichloroacetic acid (TCA) and 2, 2'-dinitro- 5, 5'-dithiodibenzoic acid (DTNB) were bought from Merck Company. 

**Animal groups**

Male Wistar rats weighing 250 ± 20 g were used in this study. The animals were kept at the animal house under controlled conditions (12h/12h light/dark cycle, 22-24^o^c temperature and appropriate humidity) with laboratory chow and water provided *ad libitum*. The study protocol using the laboratory rats complied with the general guidelines of the animal care provided by Mashhad University of Medical Sciences, Mashhad, Iran. 

The animals were randomly divided into eight groups (*n *= 8 in each group) and injected as per following protocol:

(1) Control (received only vehicle)

(2) Vehicle + PTZ (90 mg/kg)

(3 and 4) WF (25 and 100 mg/kg) + PTZ

(5 and 6) NBF (25 and 100 mg/kg) + PTZ

(7 and 8) EAF (25 and 100 mg/kg) + PTZ

The animals were treated with vehicle or different fractions 30 min before intraperitoneally (IP) injection of PTZ. The brain tissues were then removed for biochemical measurements. In our previous work, we found that hydro-alcoholic extract and fractions of *C. sativum *induced sedative/hypnotic effects at the doses of 50, 100, 200 and 400 mg/kg (16). In the current study, therefore, we used comparable doses (25-100 mg/kg) of *C. sativum *fractions to test its anticonvulsive effect.

**Plant extracts**

Here, *C. sativum* (leaves, stems, and twigs) was collected from Neyshabur area (Khorasan Razavi province, Iran). The taxonomic identity of the plant was confirmed and a voucher specimen (No. 10068) was deposited at the herbarium of school of Pharmacy (Mashhad University of Medical Sciences, Mashhad, Iran). The aerial parts of *C. sativum* were dried and then grounded to fine powder using a blender. To prepare hydroalcoholic extract, each 50 g of the powder was extracted with 300 ml ethanol-water (70/30 v/v) using a Soxhlet apparatus (Rakhshandah and Hosseini, 2006 ▶; Ghorbani et al., 2013 ▶). After 48 h, the extract was dried by a rotary vacuum evaporator (yield 30% w/w). 

For preparation of *C. sativum* fractions, each 10 g of dried extract was suspended in 300 ml distilled water, transferred to a separator funnel, and partitioned with ethyl acetate (300 mL × 6). The ethyl acetate-soluble fraction (i.e. EAF) was collected and the remaining solution was further partitioned with *n*-butanol (300 mL × 6). The NBF was also separated and the lower water-soluble layer was considered as WF (Ghorbani et al., 2015 ▶). The resulting fractions were dried on a water bath and working solutions were prepared in saline, saline containing 1% tween, and 1% DMSO for WF, EAF and NBF, respectively. The yields obtained by the extract fractionation were 78% WF, 9.6% EAF, and 12.4% NBF. 

**PTZ-induced seizures**

In order to observe ictal behavior, PTZ was injected (IP) and the animals were placed in Plexiglas arena (30 cm × 30 cm × 30 cm) on the day of the experiment. The animals were observed during a 60-min period after PTZ administration. Behavioral responses of the animals to PTZ administration were evaluated using these criteria: latency to the first minimal clonic seizure (MCS), incidence of MCS, latency to the first generalized tonic-clonic seizures (GTCS), incidence of GTCS, protection percentage against GTCS and protection percentage against mortality (Hosseini et al., 2011 ▶; Hosseini et al., 2009 ▶; Ebrahimzadeh Bideskan et al., 2011 ▶; Hosseini et al., 2012 ▶; Hosseini et al., 2013 ▶). 

For the latencies to the first MCS and GTCS, the time onset (in seconds) of first MCS (appeared as myoclonic contractions of face and forelimbs known as face and forelimb clonus) and the time onset (in seconds) of GTCS (appeared as falling, tonic contractions followed by clonic contractions of whole body) were measured. Regarding the incidence of MCS and GTCS, the number of animals per group showing MCS or GTCS was reported (Hosseini et al., 2011 ▶; Hosseini et al., 2009 ▶; Ebrahimzadeh Bideskan et al., 2011 ▶; Hosseini et al., 2012 ▶; Hosseini et al., 2013 ▶). 

**Biochemical assessment**

After behavioral study, the animals were sacrificed, and the brains were removed and dissected on an ice-cold surface and finally tissues were submitted to biochemical measurements. Total SH groups were measured using DTNB as the reagent. This reagent reacts with the SH groups to produce a yellow colored complex which has a peak absorbance at 412 nm. Briefly, 1ml Tris-EDTA buffer (pH = 8.6) was added to 50 μl brain homogenate in 1ml cuvettes and sample absorbance was read at 412 nm against Tris-EDTA buffer alone (A1). Then, 20 μl DTNB reagents (10 mM in methanol) were added to the mixture and after 15 min (stored in laboratory temperature) the sample absorbance was read again (A2). The absorbance of DTNB reagent was read as a blank (B). Total thiol concentration (mM) was calculated using the following equation (Hosseinzadeh et al., 2005 ▶; Sadeghnia et al., 2013 ▶; Khodabandehloo et al., 2013 ▶).

Total thiol concentration (mM) = (A2-A1-B) × 1.07/0.05 × 13.6

The level of malondialdehyde (MDA), as an index of lipid peroxidation, was measured. MDA reacts with TBA as a thiobarbituric acid reactive substance to produce a red colored complex which has peak absorbance at 535 nm. The TBA-TCA-HCL reagent was added to homogenate and the solution was heated in a water bath for 40 min. After cooling, the whole solutions were centrifuged at 1000 g for 10 min. Then, the absorbance was measured at 535 nm (Hosseini et al., 2012 ▶; Hosseini et al., 2013 ▶; Nassiri-Asl et al., 2013 ▶). The MDA concentration was calculated as follows: C (m) = Absorbance/(1.65 × 10^5^)

**Statistical analysis**

All data were expressed as Mean ± SEM and analyzed by using one way ANOVA followed by Tukey's post hoc comparison test. p values less than 0.05 were considered as statistically significant.

## Results

**Effect of the fractions of** ***C. sativum*** **on PTZ-induced seizures**

**Figure 1:**
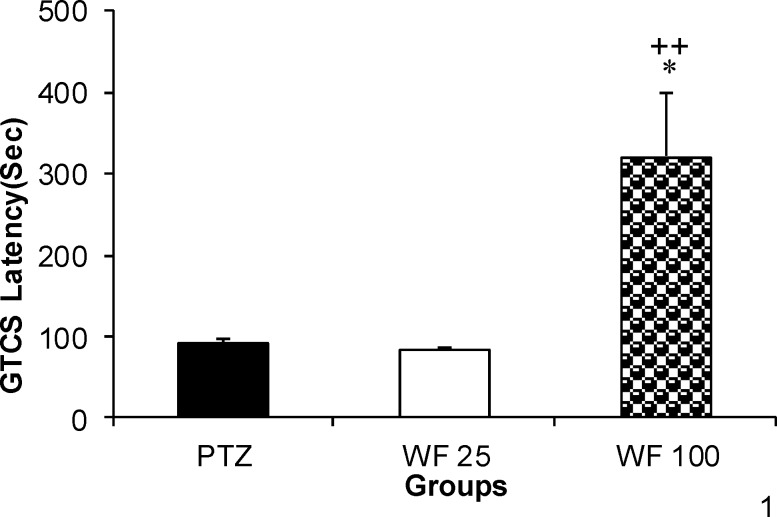
The effects of 25 and 100 mg/kg of water fraction (WF) of hydroalcoholic extract of *C. sativum *on latencies to minimal clonic seizures (MCS) (A) and generalized tonic–clonic seizures (GTCS) (B) onsets after a single injection ( 90 mg/kg ) of PTZ. PTZ: pentylenetetrazole, WF 25: water fraction 25 mg/kg, WF 100: water fraction 100 mg/kg. Data are presented as mean± SEM (n= 8 in each group).^ *^p<0.05 as compared to PTZ group,^ +^p<0.05 as compared to WF 25 group

All the animals in different treatment groups (except for the control group which did not receive PTZ) showed MCS and GTCS following PTZ administration. The WF at 25 mg/kg was not effective against MCS or GTCS (Figure 1). The WF at 100 mg/kg prolonged MCS latency, although the effect was not significant (Figure1). 

The WF at 25 mg/kg was not effective against GTCS (Figure 1); however, GTCS latency in the group treated with 100 mg/kg of WF was significantly higher than that of PTZ group (p<0.05). The effect of 100 mg/kg was significantly higher than that of 25 mg/ kg (p<0.01).

**Figure 2:**
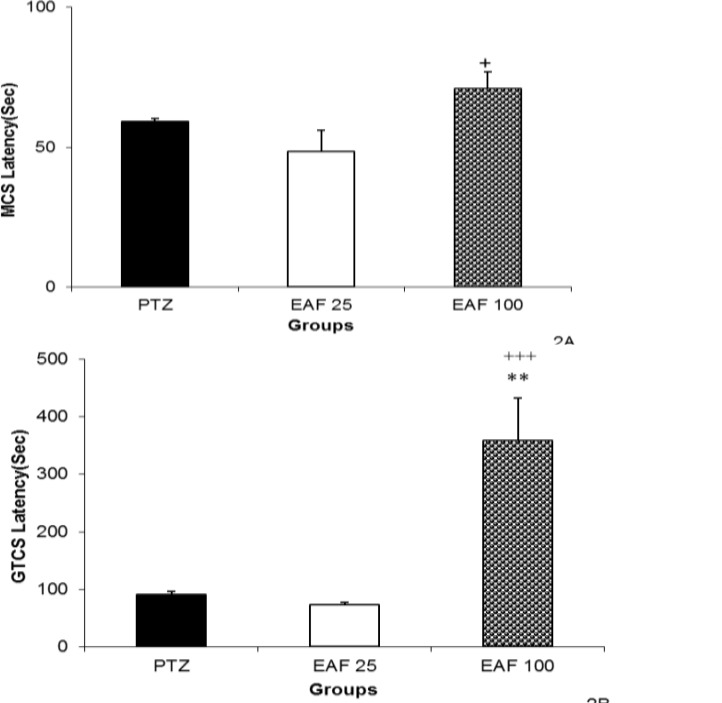
The effects of 25 and 100 mg/kg of ethyl acetate fraction (EAF) of hydroalcoholic extract of *C. sativum *on latencies to minimal clonic seizures (MCS) (A) and generalized tonic–clonic seizures (GTCS) (B) onsets after a single injection (90 mg/kg) of PTZ. PTZ: pentylenetetrazole, EAF 25: ethyl acetate fraction 25 mg/kg, EAF 100: ethyl acetate fraction 100 mg/kg. Data are presented as mean ± SEM (n= 8 in each group).^ **^p<0.05 as compared to PTZ group,^ +^p<0.05 and^ +^^++^p<0.001 as compared to EAF 25 group

Administration of 25 and 100 mg/kg of EAF had no effect on MCS latency. However, the MCS latency in the animal of EAF 100 group was longer than that of EAF 25 group (p<0.05, Figure 2). Here, 100 mg/kg of EAF prolonged the GTCS latency (p<0.01, Figure 2). 

**Figure 3:**
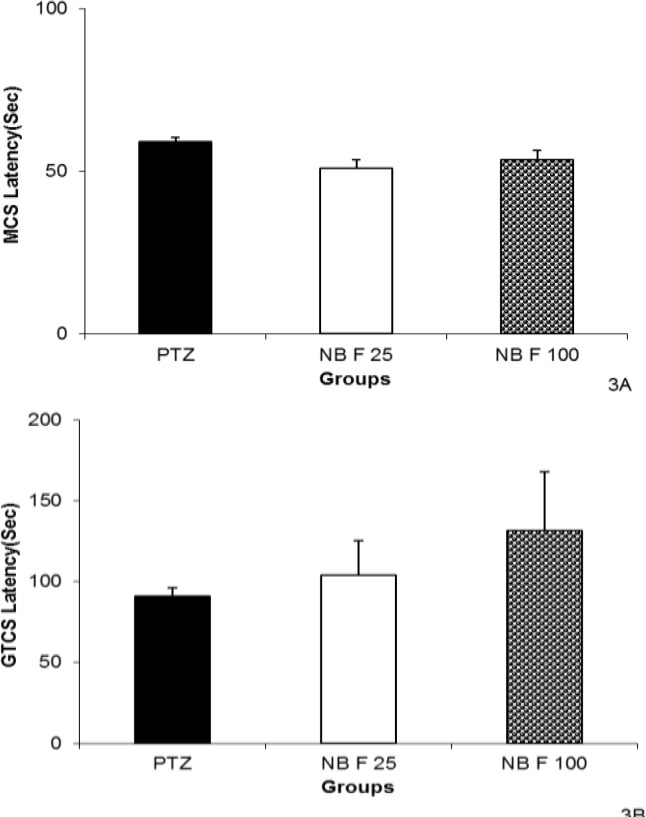
The effects of 25 and 100 mg/kg of N- butanol fraction (NBF) of hydroalcoholic extract of *C. sativum *on latencies to minimal clonic seizures (MCS) (A) and generalized tonic–clonic seizures (GTCS) (B) onsets after a single injection (90 mg/kg) of PTZ. PTZ: pentylenetetrazole, NBF 25: *n*-butanol fraction 25 mg/kg, EAF 100: *n*-butanol fraction 100 mg/kg. Data are presented as mean ± SEM (n= 8 in each group

The GTCS latency in the animals treated with 100 mg/kg of EAF was higher than that of the ones treated by 25 mg/kg (p<0.001, Figure 2). Neither 25 nor 100 mg/kg of NBF could change MCS. The GTCS latency was not affected by 25 or 100 mg of NBF (Figure 3).

**Effect of** ***C. sativum*** **on brain tissues oxidative damage**

**Figure 4:**
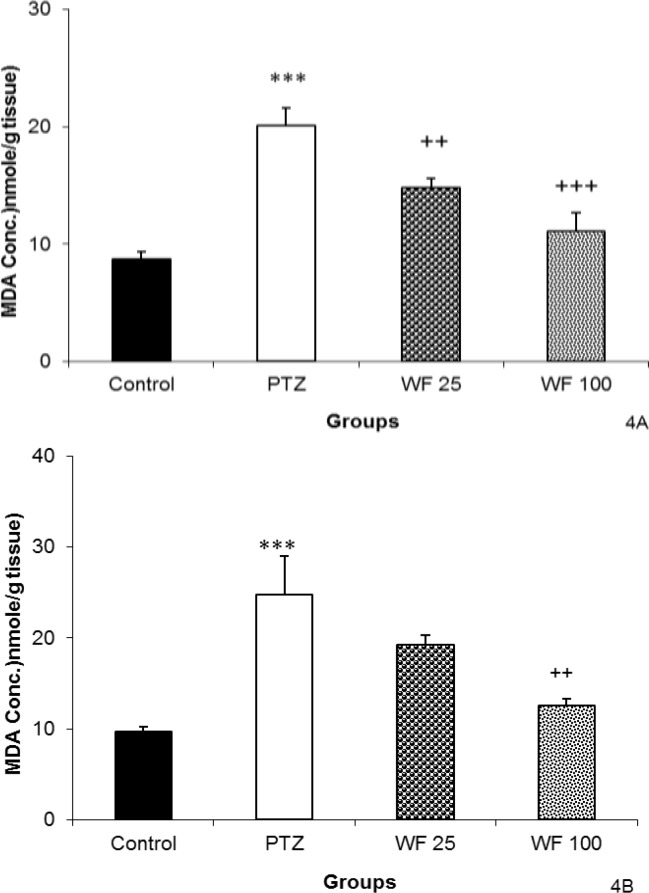
The effects of 25 and 100 mg/kg of water fraction (WF) of hydroalcoholic extract of *C. sativum *on malondialdehyde (MDA) in hippocampal (A) and cortical (B) tissues after a single injection (90 mg/kg) of PTZ. PTZ: pentylenetetrazole, WF 25: water fraction 25 mg/kg, WF 100: water fraction 100 mg/kg. Data are presented as mean± SEM (n= 8 in each group).^ ***^p<0.001 as compared to control group,^ ++^p <0.01 and ^+++^p<0.001 as compared to PTZ group. The animals of control groups received only vehicle, PTZ group was injected by PTZ, WF 25 and WF 100 groups received 25 and 100 mg/kg of water fraction before PTZ

The MDA levels in both hippocampal and cortical tissues of PTZ group were significantly higher than that of control animals (p<0.001, Figures 4A and 4B).

As shown in Figure 4A, pretreatment with both 25 and 100 mg/kg of WF resulted in a significant reduction in the free radical-mediated lipid peroxidation in hippocampal tissues as indicated by a decrease in the MDA levels (p<0.01 and p<0.001, respectively). Pretreatment with 100 mg/kg of WF also reduced MDA concentration in cortical tissues (p<0.01). However, 25 mg/kg of WF was not effective (Figure 4B).

**Figure 5:**
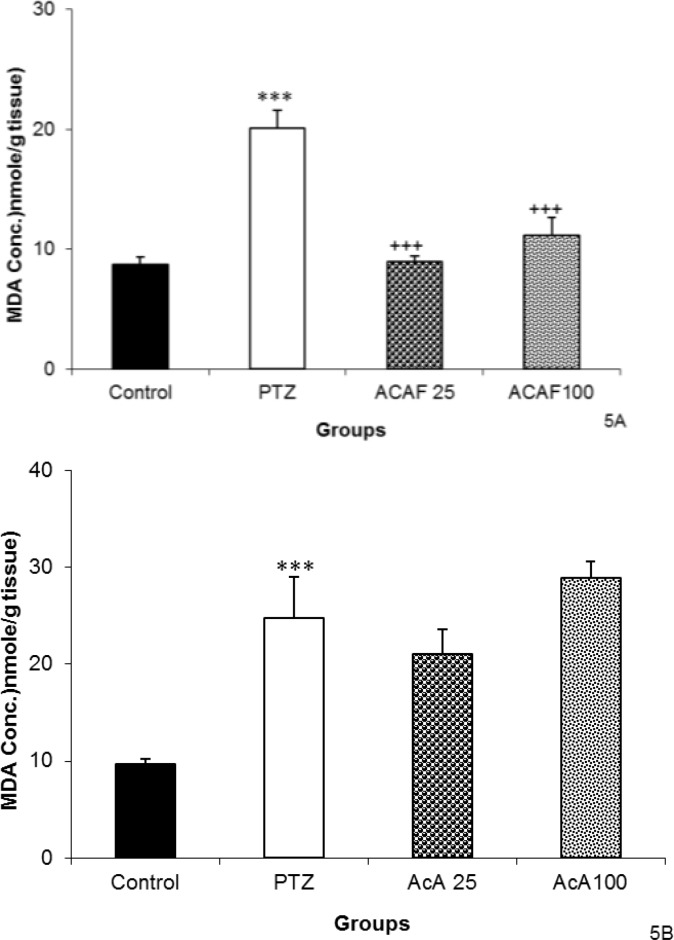
The effects of 25 and 100 mg/kg of ethyl acetate fraction (EAF) of hydroalcoholic extract of *C. sativum *on malondialdehyde (MDA) in hippocampal (A) and cortical (B) tissues after a single injection (90 mg/kg) of PTZ.

The results showed that both 25 and 100 mg/kg of the EAF reduced MDA concentration in hippocampal tissues (p<0.001 for both, Figure 5A). Neither 25 mg /kg nor 100 mg/kg of EAF could change the MDA concentration in cortical tissues (Figure 5B). 

PTZ: pentylenetetrazole, EAF 25: ethyl acetate fraction 25 mg/kg, EAF 100: ethyl acetate 100 mg/kg. Data are presented as mean± SEM (n= 8 in each group).^ ***^p<0.001 as compared to control group,^ +++^p<0.001 as compared to PTZ group. The animals of control groups received only vehicle, PTZ group was injected by PTZ, EAF 25 and EAF 100 groups received 25 and 100 mg/kg of ethyl acetate fraction before PTZ.

**Figure 6:**
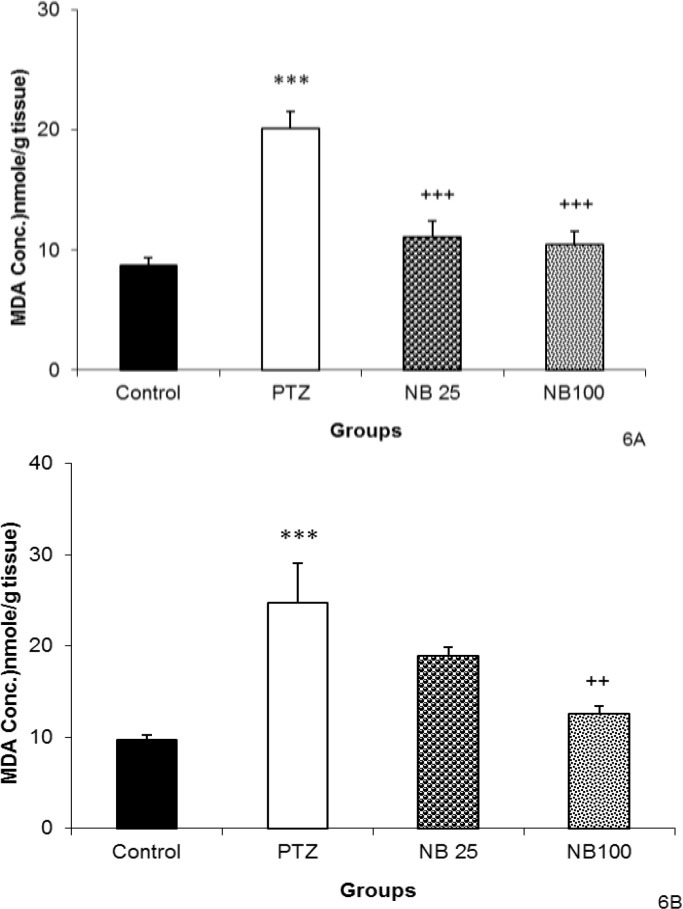
The effects of 25 and 100 mg/kg of *n*-butanol (NBF) of hydroalcoholic extract of *C. sativum *on malondialdehyde (MDA) in hippocampal (A) and cortical (B) tissues after a single injection (90 mg/kg) of PTZ

The level of hippocampal tissue MDA was reduced by pretreatment with both 25 and 100 mg/kg of NBF (p<0.001, Figure 6A). Administration of 100 mg/kg of NBF before PTZ decreased MDA concentration in cortical tissues (p<0.01) but NBF 25 mg/kg was not effective (Figure 6B). 

**Figure 7:**
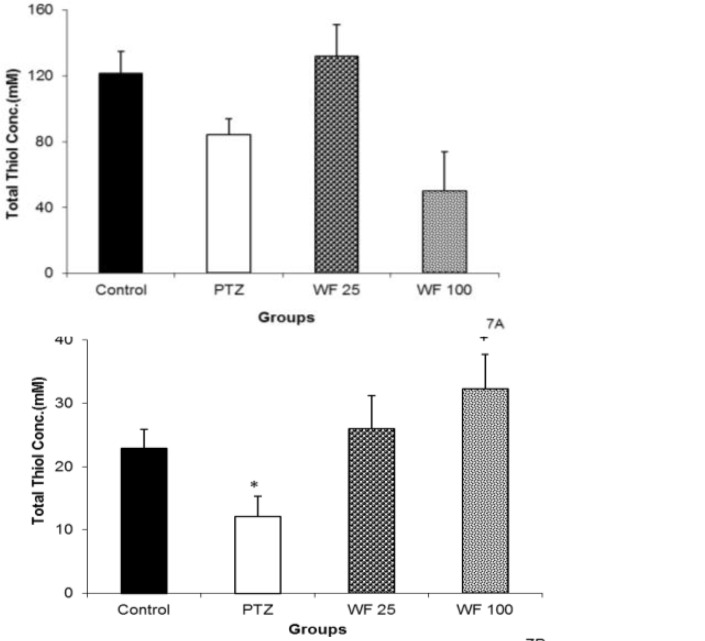
The effects of 25 and 100 mg/kg of water fraction (WF) of hydroalcoholic extract of *C. sativum *on total thiol concentration in hippocampal (A) and cortical (B) tissues after a single injection (90 mg/kg) of pentylenetetrazole (PTZ). Data are presentedd as mean±SEM (n=8).^ *^p<0.05 as compared to control group,^ +^p<0.05 as compared to PTZ group. The animals of control groups received only vehicle, PTZ group was injected by PTZ, WF 25 and WF 100 groups received 25 and 100 mg/kg of water fraction before PTZ

 Following PTZ administration, a non-significant reduction in hippocampal and a significant reduction in cortical total thiol groups were observed (p<0.05, Figures 7A and 7B). Pretreatment with 25 mg/kg of WF caused a non-significant elevation of total thiol concentration in hippocampal tissue, as compared with PTZ group (Figure 7A). There were no significant differences between WF 100 and PTZ groups when total thiol concentration of hippocampal tissue was compared (Figure 7A). However, 100 mg/kg of WF increased total thiol groups in cortical tissue (p<0.05, Figure 7B). 

**Figure 8:**
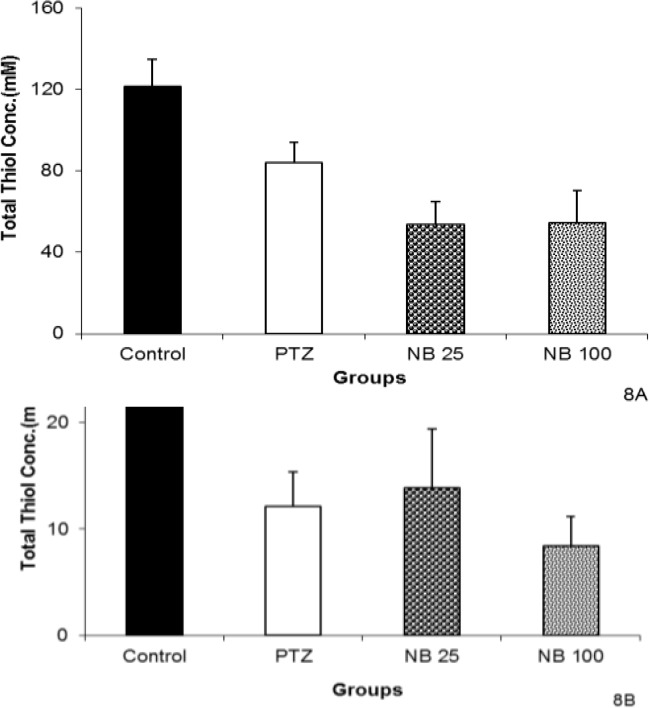
The effects of 25 and 100 mg/kg of ethyl acetate fraction (EAF) of hydroalcoholic extract of *C. sativum *on total thiol concentration in hippocampal (A) and cortical (B) tissues after a single injection (90 mg/kg) of pentylenetetrazole (PTZ). Data are presented as mean±SEM (n=8).^ *^p<0.05 as compared to control group. The animals of control groups received only vehicle, PTZ group was injected with PTZ, EAF 25 and EAF 100 groups received 25 and 100 mg/kg of ethyl acetate fraction before PTZ

Regarding EAF, both 25 and 100 mg/kg failed to change the thiol concentrations of hippocampal and cortical tissues (Figures 8A and 8B). 

**Figure 9:**
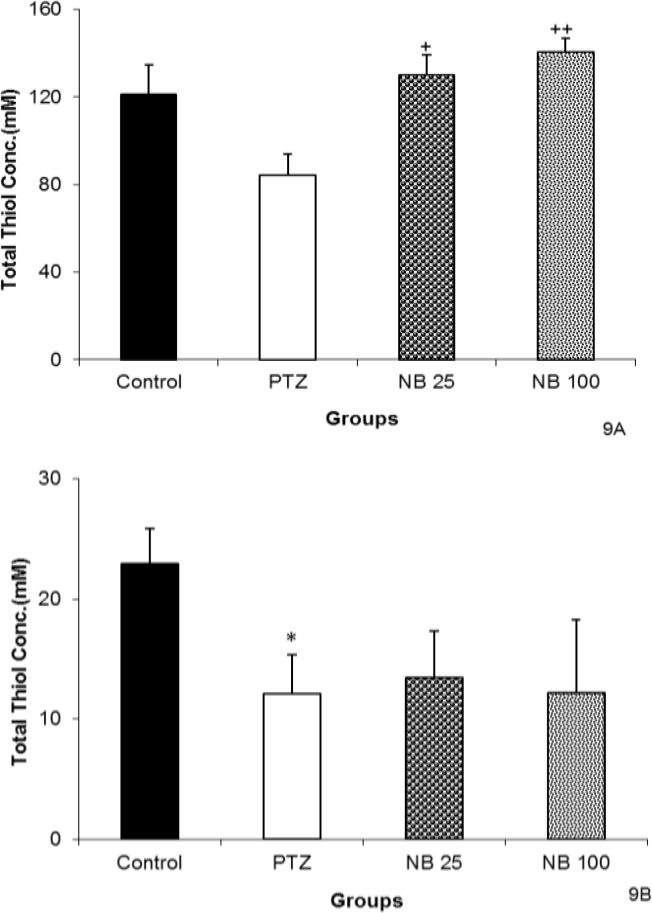
The effects of 25 and 100 mg/kg of *n*-butanol (NBF) of hydroalcoholic extract of *C. sativum *on total thiol concentration in hippocampal (A) and cortical (B) tissues after a single injection (90 mg/kg) of PTZ.

The NBF in both 25 and 100 mg/kg increased the total thiol groups in hippocampal tissues (p<0.05 and p<0.01, respectively, Figure 9A). Pretreatment with 25 or 100 mg/kg of NBF did not affect total thiol concentrations in cortical tissues compared to PTZ group (Figure 9B).

## Discussion

Oxidative stress is the basis for many neurological and neurodegenerative disorders. Previous studies demonstrated that oxidative stress plays an important role in pathogenesis of epileptic seizures (Golechha et al., 2010 ▶; Patel, 2004 ▶). Especially, an increase in free radicals level has been reported during seizures (Gupta and Briyal, 2006 ▶). Similarly, in the present study, we observed an increase in MDA levels and a reduction in total SH groups in the brain of animals subjected to PTZ-induced seizure. Increase in concentration of ROS including superoxide anions, hydroxyl radicals and hydrogen peroxide, in the brain due to epilepsy and seizure has been well documented (Sudha et al., 2001 ▶; Rodrigues et al., 2012 ▶). The elevation of ROS in the brain may lead to brain tissues oxidative damage which is followed by psychiatric as well cognitive problems such as depression, anxiety and memory loss (Costello and Delanty , 2004 ▶; Reilly et al., 2011 ▶). The decrease in the life span which has been reported in the epileptic persons may be at least in part due to oxidative damage (Maldonado et al., 2010 ▶). Oxidative stress has been also suggested as a link between aging and seizure (Liang et al., 2007 ▶). The results of the present study confirmed that lipid peroxidation in brain tissues was higher and total SH groups were lower in PTZ-treated rats than control ones. PTZ-induced seizure model has been frequently used to examine the drugs or natural compounds for potential anticonvulsant properties (Hosseini et al., 2011 ▶; Hosseini et al., 2009 ▶; Ebrahimzadeh Bideskan et al., 2011 ▶; Hosseini et al., 2012 ▶; Hosseini et al., 2013 ▶). PTZ decreases the GABA system function and the stimulation and increases the activity of glutamate neurotransmission system (White et al., 2007 ▶). The contribution of ROS in the neurotoxic effects of PTZ has been suggested (Xie et al., 2012 ▶, Liu et al., 2012 ▶) and therefore, increase in brain tissues oxidative damage which was seen in the present study is conceivable.

The animals pretreated with *C. sativum* fractions showed a reduction in lipid peroxidation and elevation in thiol concentrations. Consistent with this finding, some studies reported strong antioxidant activity for *C. sativum* (Samojlik et al ., 2010 ▶; de Almeida Melo et al., 2003 ▶; Hashim et al., 2005 ▶; Misharina and Samusenko, 2008 ▶; Sultana et al., 2010 ▶). We showed that these effects of* C. sativum* fractions are accompanied by an anti-convulsant effect as it increased both MCS and GTCS latencies. Consistent with these findings, it has been shown that several antioxidant agents such as melatonin, vineatrol, trans-resveratrol and alpha lipoic acid have anticonvulsant activity (Gupta and Briyal, 2006 ▶; Sharma et al., 2005 ▶). The antioxidant effect of *C. sativum* may also be responsible for its neuroprotective activity which was observed in our previous work (Ghorbani et al., 2011 ▶).

Recently, an anticonvulsant effect for the aqueous and ethanolic extracts of *C. sativum* seeds has been reported (Hosseinzadeh and Madanifard, 2000 ▶). The same effect was found by Emam Ghoreyshi and colleagues for hydroalcoholic and aqueous extracts and essential oil of *C. sativum* seeds (Emam Ghoreyshi and Heydari Hamedani, 2008 ▶). However, so far no pharmacological study has been done to evaluate anticonvulsant activity of aerial parts of this plant. These parts of *C. sativum* are widely consumed as vegetable all over the world, especially in the Middle East. In the present study, we showed that different fractions of *C. sativum* aerial parts are anticonvulsive and have inhibitory effects on seizure-induced oxidative stress. Therefore, consumption of aerial parts of this plant may reduce the brain tissues oxidative damage and consequently can decrease brain functional or structural disturbances which occur in epilepsy. To obtain better insight into the nature of constituents responsible for the effect of *C. sativum*, three fractions were prepared from hydroalcoholic extract: (1) WF which dissolves polar compounds and water-soluble plant constituents such as quaternary alkaloids, glycosides and tannins; (2) EAF which extract agents with intermediate polarity such as some flavonoids; (3) NBF which has non-polar agents like sterols, alkanes and some terpenoids (Ghorbani et al., 2012 ▶). Our data showed that among these *C. sativum* fractions, both WF and EAF had an anticonvulsant effect which was represented through increase of MCS and GTCS latency. Therefore, it seems that water-soluble and intermediary water-soluble constituents of *C. sativum* hydroalcoholic extract are mainly involved in the anticonvulsant activity of this plant. The presence of flavonoids such as quercetin has been reported in *C. sativum* (Kunzemann and Herrmann, 1977 ▶). Consistent with our findings, it has been shown that quercitin and other flavonoids like rutin have considerable anticonvulsant effects (Nassiri-Asl et al., 2010 ▶). The sedative and central nervous system depressant effects of flavonoids have been attributed to their affinity for central benzodiazepine receptors (Medina et al., 1997 ▶; Griebel et al., 1999 ▶; Paladini et al., 1999 ▶; Youdim et al., 2004 ▶; Kang et al., 2000). Other non-polar compounds such as isoquercitin, linalool, limonene and myrcene may also be candidates for the anticonvulsant effects of coriander (do Vale et al., 2002 ▶; Kang et al., 2000; Oganesyan et al., 2007 ▶; Picq et al., 1991 ▶; Sugawara et al., 1998 ▶). Regarding antioxidant activity of *C. sativum*, all three fractions tested in this study were able to decrease lipid peroxidation and increase thiol groups in brain tissues. Natural antioxidants have different solubilities: glutathione, ascorbic acid, phenolic compounds, and urate are water-soluble; tocopherols and carotenoids are lipid-soluble; and hydroxycinnamic acids and flavonoids are intermediatory-soluble (Eastwood, 1999 ▶). Therefore, our data show that *C. sativum* contains different antioxidant compounds. This is supported by the findings from previous works stating that aqueous extract, essential oils, and EAF of *C. sativum* have strong antioxidant activity (Samojlik et al., 2010 ▶; Satyanarayana et al., 2004 ▶; Misharina and Samusenko, 2008 ▶). Considering these antioxidant effects, potential benefits for *C. sativum* against seizures itself as well as the nervous system complications due to seizures might be suggested. Furthermore, epilepsy and seizures have been well-known to be modulated by gamma-aminobutyric acid (GABA)ergic and glutamatergic pathways in the brain. It also could be affected by several other neurotransmitters and endogenous substances such as opioids (Engelborghs et al., 2000 ▶; Ure and Perassolo., 2000 ▶ ). The same interaction with GABAergic system may be involved in the mechanisms via which coriander exerts its anti-convulsant effect. It has been shown that linalool has inhibitory effect on glutamate binding in rat cortex and therefore, induces hypnotic and anticonvulsant effects (Elisabetsky et al., 1995 ▶). Certainly, further investigations are needed to evaluate these suggestions.

The present study showed that different fractions of *C. sativum* aerial parts possess antioxidant activity in the brain. WF and EAF of this plant induced anticonvulsant effect which was represented by increase of GTCS latency. Isolation of the exact active compound(s) from these fractions may yield novel anticonvulsant agents.

## Conflict of interests

The authors declare that they have no competing interests.
